# Research on Occupational Safety, Health Management and Risk Control Technology in Coal Mines

**DOI:** 10.3390/ijerph15050868

**Published:** 2018-04-26

**Authors:** Lu-jie Zhou, Qing-gui Cao, Kai Yu, Lin-lin Wang, Hai-bin Wang

**Affiliations:** 1 College of Minging and Safety Engineering, Shandong University of Science and Technology, Qingdao 266590, China; 13969891661@163.com (L.-j.Z.); ykxfkd@163.com (K.Y.); ll860904@163.com (L.-l.W.); 2 Geting Coal Mine, Zibo Mining Group Company, Jining 272053, China; anquanwanghaibin@126.com

**Keywords:** coal mine, occupational safety and health management, risk, early warning, control, system, B/S mode

## Abstract

This paper studies the occupational safety and health management methods as well as risk control technology associated with the coal mining industry, including daily management of occupational safety and health, identification and assessment of risks, early warning and dynamic monitoring of risks, etc.; also, a B/S mode software (Geting Coal Mine, Jining, Shandong, China), i.e., Coal Mine Occupational Safety and Health Management and Risk Control System, is developed to attain the aforementioned objectives, namely promoting the coal mine occupational safety and health management based on early warning and dynamic monitoring of risks. Furthermore, the practical effectiveness and the associated pattern for applying this software package to coal mining is analyzed. The study indicates that the presently developed coal mine occupational safety and health management and risk control technology and the associated software can support the occupational safety and health management efforts in coal mines in a standardized and effective manner. It can also control the accident risks scientifically and effectively; its effective implementation can further improve the coal mine occupational safety and health management mechanism, and further enhance the risk management approaches. Besides, its implementation indicates that the occupational safety and health management and risk control technology has been established based on a benign cycle involving dynamic feedback and scientific development, which can provide a reliable assurance to the safe operation of coal mines.

## 1. Introduction

What accompanies the nationwide industrialization in China is the exacerbation of work injuries and occupational diseases. As such, occupational safety and health management are getting more and more attention. Statistical data from a broad spectrum of sources have indicated that human factors (i.e., deliberate violations, management failures, and faulty designs) account for 97.67% of coal mine accidents in China [1]. Mining enterprises, especially those focusing on coal mining, need to lay strong emphasis on the occupational safety and health management (risk management in particular) during the production due to their exotic operating environment, complicated operating conditions, and numerous risky factors. At the same time, advancements in science and technology have created more and more opportunities in a diverse manner to help enhance the health of the operators [2,3,4,5].

Since the 1990s, some developed countries have taken the lead in implementing the OSHMS system [6,7]; China has carried out the research and implementation activities concerning OHSMS following the global trend. In 2001, China’s AQSIQ (Administration of Quality Supervision, Inspection and Quarantine) issued the “Occupational Health and Safety Management System Standard” (GB/T 28001-2001), which was in turn revised and republished in 2011 (GB/T 28001-2011) [8]. To drive the occupational health and safety management in the coal mining industry, the State Administration of Production Safety Supervision and Administration issued the “Temporary Regulation on Supervision and Management of Occupational Health in Fields” and the “Prevention and Treatment of Occupational Diseases in Coal Mining Fields” in 2009 and 2015, respectively. In 2011, the “Instructions on Promoting Occupational Health” was issued in 2011 [9], putting forward a suite of clear standards and stringent requirements. Meanwhile, China is also working to ensure that the occupational safety and health as well as human factors are all integrated into the broad framework of sustainable development [10].

According to the national requirements and the practical needs for ensuring safe operations in enterprises, various enterprises, e.g., those in the coal mining industry, have managed to establish an occupational safety and health management system, and carry out the occupational safety and health management activities accordingly, leading to decent results [11,12]. However, according to the analyses of coal mine occupational safety and health management practices over recent years, an array of issues are identified: (1) although much attention has been given to the establishment and certification of occupational safety and health management system, the daily activities are not conducted strictly following the established system; (2) although the occupational safety and health management system is macroscopically well-established, it lacks practicality from a microscopic perspective; (3) during the safety management, only the directly responsible personnel are subject to disciplinary actions, while those indirectly involved in the accidents are not held accountable; (4) the risk control effects are subpar, with personal injuries occurring frequently; and (5) effective communication is inhibited, while occupational safety and health management lacks scientific and clear procedures and a suite of user-friendly and practically valuable software packages for providing support.

The aforementioned issues are due to the lack of practical occupational safety and health management methods and the associated operating procedures, along with the corresponding software and network supports [13]. Aiming to address the actual requirements of coal mining enterprises, the present research focuses on the methods of occupational safety and health management and risk control technology applicable in coal mining enterprises. In addition, a software system is developed to support the coal mine occupational safety and health management application so as to scientifically, effectively, and canonically carry out the occupational safety and health management in coal mines by fully leveraging the information and network technologies, leading to enhanced occupational safety, health management and risk control standards.

## 2. Coal Mine Occupational Safety, Health Management and Risk Control 

The occupational safety and health management in coal mine enterprises, as built upon the execution of the aforementioned standards and instructions, should focus on control of the entire process pertaining to the occupational safety and health management, overall improvement of occupational safety and health behaviors [14,15], and the enhancement of risk identification and control [16,17]. According to this understanding, by collectively taking into consideration the actual needs of coal mining enterprises and early stage fundamental research results [12,18], the present study carries out a systematic and in-depth research work. In addition, the occupational safety and health management and risk control technology has been implemented in the Ge Ting coal mine and the Tang Kou coal mine, as shown below:

### 2.1. The Establishment of Occupational Safety and Health Management System, and the Reinforcement and Improvement of Daily Activities Concerning Occupational Safety and Health

Coal mining enterprises should establish an occupational safety and health management system that names the mine director as the topmost leader, and establishes a leadership team for mine occupational safety and health management. Under this leadership team is the occupational safety and health management office spearheaded by the Director of Safety Supervision concurrently serving as the director of office, focusing on organizing and implementing the occupational safety and health management. The relevant daily activities include (1) the promotion and training concerning occupational safety and health rules as well as the preventive measures of occupational hazards; (2) detection and treatment of occupational hazards (dust, noise, toxic gases, etc.); (3) distribution and use of personal protective equipment; (4) prevention of occupational diseases; and (5) monitoring of employee health, etc. It is critical to ensure the effective implementation of these efforts, with monthly evaluation of various activities for correcting problematic items in a timely manner. 

### 2.2. Timely Collection and Canonical Treatment of Occupational Safety and Health Management Information

In order to carry out occupational safety and health management scientifically and effectively, the occupational safety and health management information should be collected in a timely manner and handled properly. The most essential part of the collected information of occupational safety and health management is related to the real-time risk information during the process of coal mine production, which also includes the safety information concerning daily activities of occupational safety and health management. The real-time risk information associated with coal mine operations is collected by dedicated safety inspectors and various management personnel in the field, which is conducted in parallel with the risk identification.

The occupational safety and health management information should be obtained in a timely manner, classified clearly, and processed properly. Subsequently, the processed information is entered into the computer, and uploaded to the intranet server for storage and management.

### 2.3. Risk Identification and Assessment

The risk information of coal mine production concerns information of hazards, potential accidents, and human misconducts. The present manuscript will introduce the risk identification and assessment methods in terms of potential accidents.

#### 2.3.1. Risk Identification

The risk identification is generally conducted based on empirical analysis or system safety analysis methods [19]. As described above, the dedicated safety inspectors and management staff collect real-time risk information, and conduct risk identification based on their own empirical experiences; for more serious risks, expert consultancy is leveraged to identify risks. The system safety analysis, on the other hand, uses analysis concerning incident types and impacts, safety checklists, incident tree analysis, and accident tree analysis to achieve a comprehensive and systematic identification over risk information. As described above, during the coal mining operations, risk information is collected in real-time by dedicated safety inspectors and various management staff, followed by risk identification exercises: at general sites, experiences are leveraged to identify risks; at those important or critical sites, e.g., coal mining face, excavation face, and blasting operation posts, dedicated safety checklists should be used to ensure safe operations and obtain risk identification outcomes. 

Risk identification results are recorded in Risk Identification and Control Record Table. As listed in Table 1, Risk Identification and Control Record Table is actually a professional safety checklist, and mainly includes time, location, risk content, risk grade, and the related treatment advice. The risk grading process is detailed in Table 2, the risk content refers to the specific information recorded during the risk identification, and the treatment advice refers to the detailed treatment measures of the corresponding risks, which should be formulated based on the practical risk content and grade (as listed in Table 2).

In Table 1, the nature of risk refers to the source of danger, hidden danger, and humans’ behavioral risks; if the risk is determined as hidden danger, the type should also be determined. According to the statistics and classification standards in The Specifications of Injured and Fatal Accident Report and Statistics for the Enterprises in Coal Mine Industry, the casualty accidents in the coal mine industry can include roof, gas, electromechanical, transport, blasting, fire, and flooding accidents, among others. 

#### 2.3.2. Risk Assessment

The developed science and technology is increasingly able to characterize the contributions of various risks and interactions in-between, along with their effects on human health.

Some common risk evaluation methods include risk matrix method, operating condition hazard evaluation method, and special coal mine safety evaluation method [20,21]. The Department of Labor of the People’s Republic of China formulated The Administrative Provisions of Latent Danger of Major Accident, which came into effect on 1 October 1995. By referring to the risk matrix method, this study classifies the latent risks of mine accidents into three grades according to the actual requirements in mine safety management, seriousness of hidden dangers, and the related level of difficulty in treatment, as reflected by the classification results listed in Table 2. 

As shown in Table 2, in the case where type-A (significant) latent risks are identified, one should warn the whole mine, and report the risk to the group company for immediate treatment; additionally, the related operators should receive education, training and strict examination; for type-B (serious) latent risks, one should issue warning to the whole mine and ask the personnel concerned to address the issue within a specified time limit; if the latent risks are classified as type-C (moderate), one should remind the related personnel in the unit in charge, and ask the personnel concerned to address the issue immediately. 

### 2.4. Risk Early Warning and Dynamic Monitoring

Risk early warning aims to monitor the development trend, evaluate the deviation degree of the risk state from the critical pre-warning value, send out the early warning signal, and adopt the pre-controlling measures in advance [22,23,24]. Early warning and dynamic monitoring are required in coal mine production so as to ensure that the risk can be controlled below the acceptable level. 

#### 2.4.1. Risk Early Warning

This study conducts early warning or caution in a targeted way according to the actual requirements in the prevention of accidents as well as the practical condition of risk grade and treatment.

For type-A risks with extremely serious damages or great management difficulty, early warning information should be released in the mine and the risks should also be reported to the group. 

For type-B risks with serious damages or management difficulty, early warning information should be released in public in the whole mine and the mine should be treated within the prescribed timeframe. If these risks are not successfully treated near the deadline, the whole mine should be informed. 

For type-C risks with moderate damages or less effects on safe production, one should prompt the units in charge to take measures immediately. 

The early warning of coal mine accident risks and occupational safety and health management are mainly achieved through the presently designed application software, i.e., Coal Mine Occupational Safety, Health Management and Risk Control System. In this system, the risk early warning is implemented through the scrolling information, flashing menus, and pop-up messages in the system.

#### 2.4.2. Risk Dynamic Monitoring

Real-time dynamic monitoring of risk is decently achieved through the “Mine Occupational Safety and Health Management and Risk Control System”, which includes the following components:

(1) Risk management dynamic monitoring, which aims to monitor the risk management status in a real-time and dynamic manner, accompanied by automatic treatment and timely issuance of control commands. The risk level is quantified through an internal risk assessment algorithm, with the relevant attributes analyzed. For instance, if an accident is left untreated or fails to receive adequate treatment within the allocated time, the system would send out warning messages, requesting an immediate investigation of the causes along with the proposal for mitigation strategies.
(2) In practice, the system compiles and issues mitigation notifications and re-inspection forms concerning latent risks so as to urge the units in charge to take care of the risks within a certain timeframe as well as re-inspecting the treatment outcomes in accordance with the re-inspection checklist so as to deliver dynamic monitoring.
(3) Dynamic monitoring of production sites. The system can automatically monitor and manage the risk situations at various production sites. Specifically, in the case that a production site exhibits accident risks, especially those serious ones, the system would issue warning and reference information, asking for timely reaction.
(4) Dynamic monitoring of responsible units. The system can conduct automatic surveillance and analysis on various units associated with coal mining enterprises so as to warn the units in charge in a timely manner to complete the risk mitigation work on time; meanwhile, the system monitors the risk treatment status of various units, and sends warning messages according to the aforementioned rules so as to urge various units to proactively and effectively control risks and prevent the occurrence of accidents.

In addition to this dynamic risks monitoring, one also needs to analyze the additional information on occupational safety and health management in coal mines so as to allow dynamic monitoring and treatment.

## 3. Development of Occupational Safety, Health Management and Risk Control System for Coal Mines

### 3.1. System Requirements Analysis and Modular Structure

**Figure 1:**
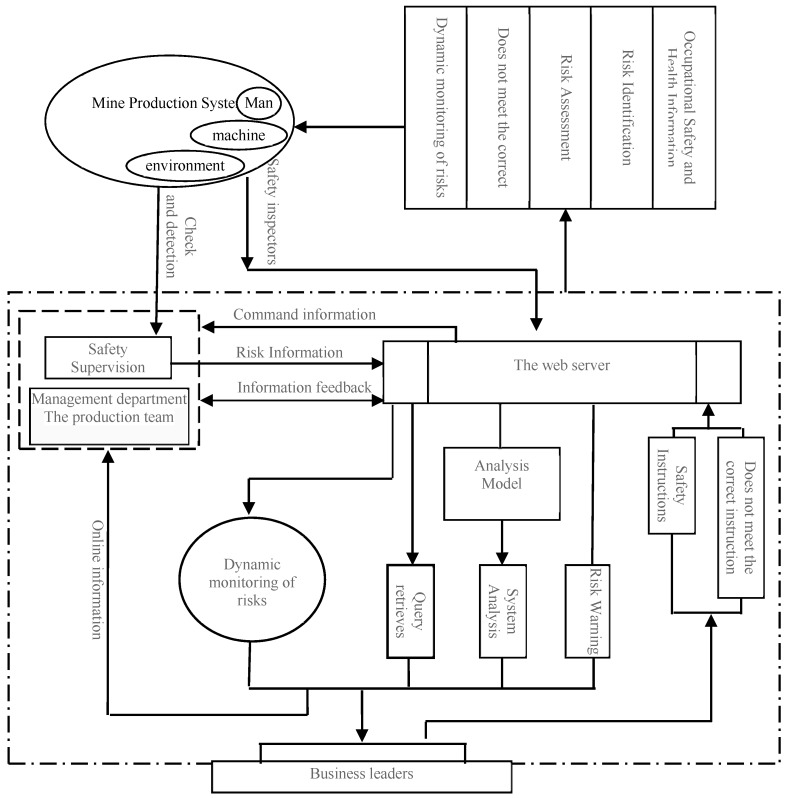
System flowchart for coal mine occupational safety, health management and risk control.

As mentioned above, throughout the whole process of coal mine occupational safety and health management [25,26], there exists a need to propagate and process a large amount of information (particularly the risk-related information) and conduct risk early warning and dynamic monitoring. These issues can be best resolved by computers and computer networks. In addition, the application of computer network to drive the occupational safety and health management is in line with the needs for informatization and modernization of coal mining enterprises [27,28]. Therefore, the development of online occupational safety and health management and risk control application software is demanded by the effort to promote coal mine safety. The scholars in this research area have also pointed out that the early warning systems can assist in identifying, predicting, and assessing the work-related injuries [29]. Through a comparative analysis, the present study develops a B/S mode application software, namely the Mine Occupational Safety and Health Management and Risk Control System. The system, running on the regional network (i.e., intranet), conducts various tasks associated with occupational safety, health management and risk control in coal mining enterprises, with the workflow shown in Figure 1.

This system manages to implement all functions needed for coal mine occupational safety, health management and risk control, with each function delivered by the corresponding module. According to the above analysis and by taking into consideration the practical needs associated with coal mine occupational safety, health management and risk control, seven modules are designed, such as daily management of occupational safety and health, risk warning, dynamic monitoring of risks, and information retrieval analysis; the system database [30] is built based upon SQL Server 2008 (Microsoft, Redmond, WA, USA).

### 3.2. System Development, Functions, and Application Methods

#### 3.2.1. System Development Method and Operating Environment

Active Server Pages 3 & ADO; VBScript & JavaScript.

The system design here is based on the prototyping design pattern along with the object-oriented method, with the design approach, tools and key techniques [31] listed below:

Active Server Pages 3 & ADO; VBScript & JavaScript
The server operating environment: Windows 2003 Server Chinese version.
Operating environment of client workstation:
Operating system: Windows 7, Windows 8, Windows XP or Windows Vista.
Browser: Microsoft IE 6.0 or later,
Internet: reliably connected with the enterprise LAN.

#### 3.2.2. System Functions and Application Methods

Through system design, programmatic implementation, and software testing, we have completed the development of the coal mine occupational safety, health management and risk control system. The system operates on the coal mine enterprise LAN (Local Area Network), allowing the input and editing of information tied to the occupational safety and health management, daily management for occupational safety and health, risk warning, risk dynamic monitoring, information retrieval, command issuance, and system maintenance. The applications of these functions are illustrated below with examples: 

##### System Startup and Homepage

**Figure 2:**
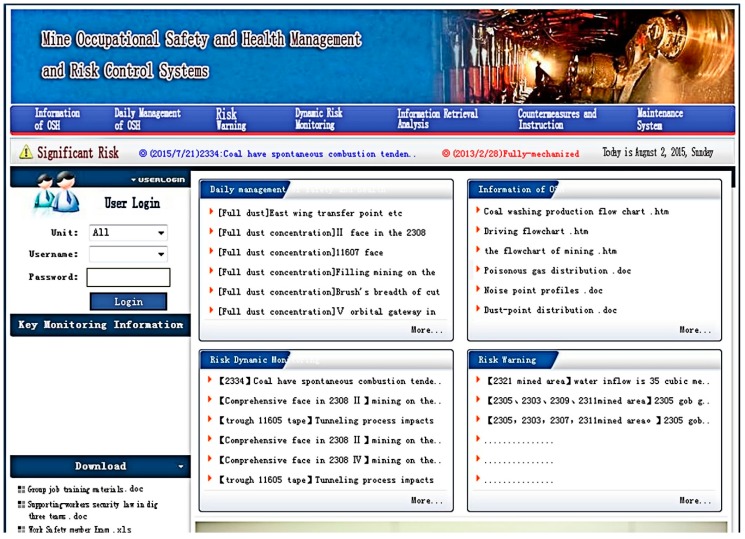
Homepage of coal mine occupational safety, health management and risk control system.

The logon page of the system is oshms.asp. Open the internet browser on a client terminal machine connected to the internet, and enter the address of system homepage to pull up the logon page and start the system. Upon the completion of logon, the homepage is shown in Figure 2.

##### Daily Management of Occupational Safety and Health

Daily management of occupational safety and health includes five functions, e.g., correction of inconsistency, performance evaluation, and monitoring of employee health, etc. One needs to follow the instructions to complete the operation.

##### Risk Early Warning

**Figure 3:**
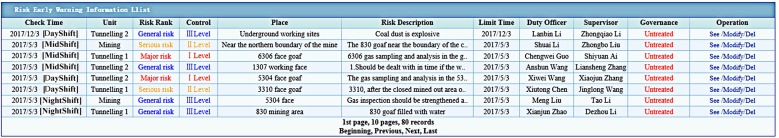
Risk early warning information list.

To be specific, risk early warning includes three functions, namely, general risk warning, early warning of serious risk, and caution of major risk. As shown in Figure 2, both caution information and major risks are displayed on the system homepage in a continuous loop on the scroll bar. As shown in Figure 3, early warning and reminder signal should be clearly sent out to the relevant users or can be inquired or received via the menu of “Risk Early Warning”. For clearly displaying the query results, the risks at different grades are marked in different colors; specifically, major risks are marked in red, serious risks are marked in yellow, and general risks are marked in blue. In addition, the untreated risks are marked as “Untreated” in red. 

##### Dynamic Monitoring of Risk

**Figure 4:**
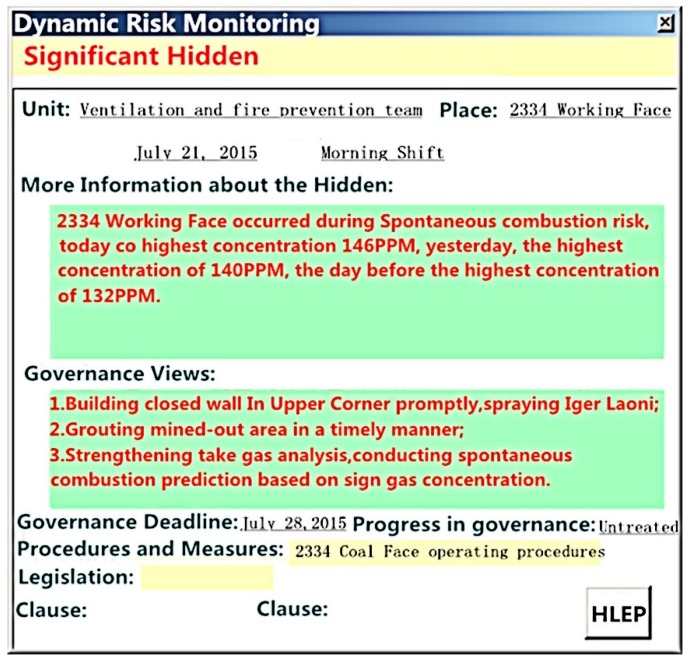
Information concerning dynamic monitoring of a major hidden risk.

This includes three specific functions, e.g., risk management monitoring, monitoring of production sites, and monitoring of responsible unit. These functions are all carried out in accordance with the principles and methods of the risk dynamic monitoring detailed previously. For example, the dynamic monitoring information associated with a major hidden risk is as shown in Figure 4.

## 4. Application and Analysis of Occupational Safety and Health Management and Risk Control Technology in Coal Mine Enterprises

### 4.1. The Implementation of Occupational Safety and Health Management and Risk Control Technology System in Coal Enterprises

Based on aforementioned mine occupational safety, health management and risk control technology, a supporting application software, namely, Coal Mine Occupational Safety, Health Management and Risk Control System, is developed. After the test in Geting Mine owned by Zibo Mining Group in July 2014, the software is put into use. Afterwards, the software was used in Shandong Tangkou Coal Mining Company, starting in March 2016. The implementation of this technology system has gained favorable results, which can ensure timeliness, normalization and accuracy of occupational safety and health management. This system can analyze occupational safety and health management condition in an enterprise in a timely manner, and find and rectify inappropriate phenomena in time. Especially, risk early warning and dynamic control technology can achieve real-time monitoring and dynamic control, and guide risk management, thereby effectively enhancing risk management efficiency and risk control effect and guaranteeing the enterprise’s high-quality, stable and safe production. 

After the implementation of this technology system, all the workers in Geting Coal Mine exhibit increasing safety awareness and the managers at all levels show enhanced responsibility for safe production; accordingly, the latent risks and three violations (namely, illegal command, illegal operation and violation of labor discipline) can be found faster and more frequently, which can also be addressed more rapidly, comprehensively and thoroughly; even some problems difficult to identify in the past can be identified and addressed timely. Both the numbers and seriousness degrees of hidden dangers and three violations dropped significantly. Table 3 lists the related data during the period from July 2013 to June 2017. After the implementation in Shandong Tangkou Coal Industry Co., Ltd. (Jining, China), latent risks and three violations decreased obviously in the second half of 2016 and some serious problems were addressed in real time. Owing to the implementation of this technology system in Geting Mine and Shandong Tangkou Coal Industry Co., Ltd., no major accidents with deaths and serious injuries were reported, and safe production level in the mines underwent stable and sustainable improvement.

**Figure 5:**
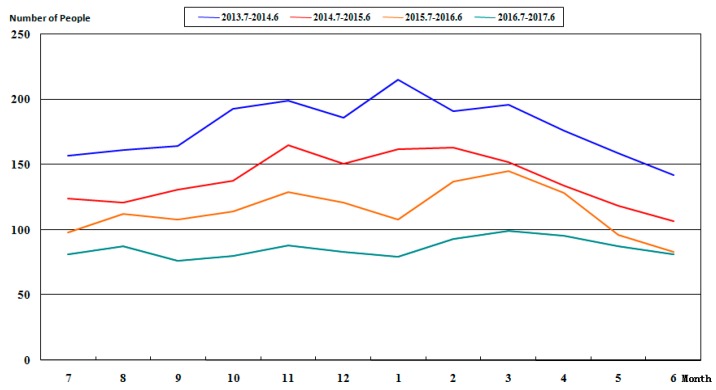
Variation tendency of the number of people under latent risks from July 2013 to June 2017.

**Figure 6:**
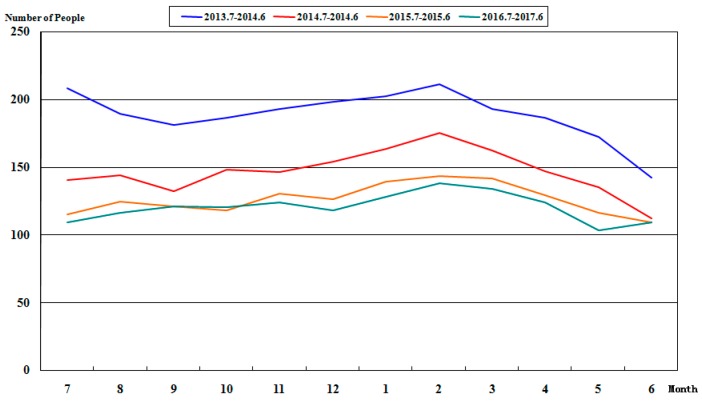
Variation tendency of the number of people under three violations from July 2013 to June 2017.

According to the statistical data in Table 3, the numbers of people under latent risks and three violations in Ge Ting Mine during the period from July 2013 to June 2017 are plotted for comparison, as shown in Figure 5 and Figure 6. It can be observed that, after the use of this system since July 2013, the number of people under latent risks and three violations drops. The blue line in Figure 5 displays the number of people under latent risks from July 2013 to June 2014; using the data as the reference, the numbers of people under latent risks from July 2014 to June 2015, from July 2015 to June 2016, from July 2016 to June 2017, are all lower than the number before the use of this system. The blue line in Figure 6 displays the number of people under three violations during the period from July 2013 to June 2014. Compared with this value, the numbers of people under three violations from July 2014 to June 2015, from July 2015 to June 2016, and from July 2016 to June 2017, are lower. In particular, the monthly numbers of people during the period from July 2013 to June 2016 drop year by year; however, the data from September 2016 to October 2016 were increased compared with the data in the same period of last year. Then, the enterprise leaders adjusted the governance method in a timely manner and the number of people with three violations returned to normal. 

### 4.2. Analysis on the Application of Occupational Safety and Health Management and Risk Control Technology in Coal Mine Enterprises

The results of this paper and its application in coal mine enterprises are analyzed. In view of the occupational safety and health management and risk control technology in coal mines, the following rules are applied to the coal mine enterprises.

(1) The implementation of coal mine occupational safety and health management and risk control technology allows the coal mine occupational safety and health management mechanism to be further improved, and the risk identification and control quality to be further bolstered.

Through the implementation of occupational safety and health management and risk control technology system, the coal mine enterprise occupational safety and health management mechanism becomes further improved and standardized, the coal mine enterprise risk identification and control level is clearly improved, and the application of coal mine occupational safety and health management and risk control system becomes more scientific, standardized and convenient.

(2) The improvement of occupational safety and health management mechanism improves the safety practice of coal mine workers.

The coal mine enterprise should establish a perfect occupational safety and health management system and take effective implementation in practical safety production, which can significantly enhance the workers’ safety awareness and strengthen the responsibility of safety production as well as the consciousness of observing disciplines and operating in a standardized way. Moreover, the workers should pay attention to vocational study and improve safety skills. 

(3) Promoting the safety awareness among workers clearly enhances the safety standard of the worker behaviors.

Improve the safety of miners, encourage workers to perform the occupational safety and health management practices more consciously, enforce the rules and operating procedures strictly, focus on identification and control of risks, and strive to improve the safe behaviors at work.

(4) Promoting the safe behaviors among workers clearly inhibits the occurrence of “three violations” and other unsafe behaviors

The enhancement of occupational safety behavior level directly leads to a significant decrease of three violation behaviors (see Table 3). Meanwhile, the incident rate of some unsafe behaviors mainly including inattention, wrong operating position and improper walking and staying also obviously dropped. 

(5) Promoting safety awareness among workers markedly expedites the handling efficiency of hidden risks and the processing quality. 

Promoting the safety awareness among workers makes the managers at all levels and all the staff more actively identify, and more effectively deal with the potential risks, leading to a gradual reduction of potential accidents (see Table 3). Meanwhile, the efficiency of identifying risks and the quality of risk handling are greatly improved.

(6) Occupational safety and health management and risk control should establish a perfect dynamic feedback mechanism. To this end, the coal mine occupational safety and health management and risk control system provides an advanced and practical technical means.

Coal mine occupational safety and health management and risk control should be carried out on a dynamic and comprehensive basis, and a sensitive and perfect dynamic feedback mechanism should be established. In view of the development and evolution of coal mine production, it is necessary to identify accident risks, implement risk early warning and risk dynamic monitoring, timely check and evaluate the effect of risk management measures in a timely manner, feedback relevant information, and conduct secondary treatment of non-compliance. The coal mine occupational safety and health management and risk control system provides an advanced technology system to achieve these goals. Such a system can be implemented in a convenient and standard manner with the aid of computer networks (LAN or intranet). 

It should be noted that, in addition to the need for development of further scientific and technical methods, there are also ethical, legal, social, and political considerations. While these issues are beyond the scope of this article, their importance cannot be underestimated.

## 5. Conclusions

Coal mine occupational safety and health management should be based on scientific methods and means, and make full use of information technology and network technology. This paper studies the coal mine occupational safety and health management and risk control technology, and correspondingly designs an online coal mine occupational safety and health management and risk control system. Moreover, the applicability and effectiveness of this system to coal mining enterprises are analyzed. The research shows that the coal mine occupational safety and health management and risk control technology system and its supporting software can scientifically and effectively drive coal mine occupational safety and health management, while scientifically and effectively reducing the accident risks so as to provide safety assurance to coal mining operations. The implementation of occupational safety and health management and risk control technology in coal mine can further improve the coal mine occupational safety and health management mechanism and the risk control effectiveness can be further enhanced. As a result, the occupational safety and health management and risk control technology can be carried out based on a benign cycle with dynamic feedback and scientific development.

## Figures and Tables

**Table 1:** Risk identification and control record table.

Inspector: Section in charge: Date: year month day Work shift:									
**Production Sites**	**Inspection Time**	**Risk Content**	**Risk Nature**	**Risk Type**	**Risk Grade**	**Treatment Advice**	**Treatment Deadline**	**Status**	**Remark**
									
									
									

**Table 2:** Rating of coal mine accidents.

Accident Rating	Classification Criteria
A—significant	May impose very serious harm or treatment is difficult, need to engage the company and the higher authorities to solve problems collectively.
B—serious	May impose serious harm or is associated with significant workload; needs to be resolved within the prescribed deadline.
C—moderate	Have an impact on safety; can be immediately resolved by the involved team or business unit.

**Table 3:** Statistics of latent risks and “three violations” in Geting Mine from July 2013 to June 2017.

		Year	Hidden Danger				Three Violations			
	Number		2013–2014	2014–2015	2015–2016	2016–2017	2013–2014	2014–2015	2015–2016	2016–2017
Month										
7			157	124	98	81	208	140	115	109
8			161	121	112	87	189	144	124	116
9			164	131	108	76	181	132	121	121
10			193	138	114	80	186	148	118	120
11			199	165	129	88	193	146	130	124
12			186	151	121	83	198	154	126	118
1			215	162	108	79	202	163	139	128
2			191	163	137	93	211	175	143	138
3			196	152	145	99	193	162	141	134
4			176	134	128	95	186	147	129	124
5			159	119	96	87	172	135	116	103
6			142	107	83	81	142	112	109	109
